# Distinct functions of wild-type and R273H mutant Δ133p53α differentially regulate glioblastoma aggressiveness and therapy-induced senescence

**DOI:** 10.1038/s41419-024-06769-5

**Published:** 2024-06-27

**Authors:** Sebastien M. Joruiz, Natalia Von Muhlinen, Izumi Horikawa, Mark R. Gilbert, Curtis C. Harris

**Affiliations:** 1 Laboratory of Human Carcinogenesis, Center for Cancer Research, National Cancer Institute, National Institute of Health, Bethesda, MD USA; 2 Neuro-Oncology Branch, Center for Cancer Research, National Cancer Institute, National Institutes of Health, Bethesda, MD USA

**Keywords:** Oncogenes, Mechanisms of disease, Cell growth, Cell invasion, Senescence

## Abstract

Despite being mutated in 92% of *TP53* mutant cancers, how mutations on p53 isoforms affect their activities remain largely unknown. Therefore, exploring the effect of mutations on p53 isoforms activities is a critical, albeit unexplored area in the p53 field. In this article, we report for the first time a mutant Δ133p53α-specific pathway which increases IL4I1 and IDO1 expression and activates AHR, a tumor-promoting mechanism. Accordingly, while WT Δ133p53α reduces apoptosis to promote DNA repair, mutant R273H also reduces apoptosis but fails to maintain genomic stability, increasing the risks of accumulation of mutations and tumor’s deriving towards a more aggressive phenotype. Furthermore, using 2D and 3D spheroids culture, we show that WT Δ133p53α reduces cell proliferation, EMT, and invasion, while the mutant Δ133p53α R273H enhances all three processes, confirming its oncogenic potential and strongly suggesting a similar in vivo activity. Importantly, the effects on cell growth and invasion are independent of mutant full-length p53α, indicating that these activities are actively carried by mutant Δ133p53α R273H. Furthermore, both WT and mutant Δ133p53α reduce cellular senescence in a senescence inducer-dependent manner (temozolomide or radiation) because they regulate different senescence-associated target genes. Hence, WT Δ133p53α rescues temozolomide-induced but not radiation-induced senescence, while mutant Δ133p53α R273H rescues radiation-induced but not temozolomide-induced senescence. Lastly, we determined that IL4I1, IDO1, and AHR are significantly higher in GBMs compared to low-grade gliomas. Importantly, high expression of all three genes in LGG and IL4I1 in GBM is significantly associated with poorer patients’ survival, confirming the clinical relevance of this pathway in glioblastomas. These data show that, compared to WT Δ133p53α, R273H mutation reorientates its activities toward carcinogenesis and activates the oncogenic IL4I1/IDO1/AHR pathway, a potential prognostic marker and therapeutic target in GBM by combining drugs specifically modulating Δ133p53α expression and IDO1/Il4I1/AHR inhibitors.

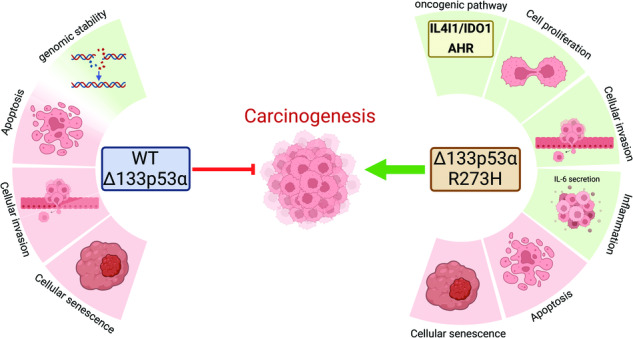

## Introduction

*TP53*, the most frequently mutated gene in human cancers [1], naturally expresses at least 12 different p53 isoforms, which share a central domain [2]. Therefore, in 92% of *TP53* mutant cancers, all p53 isoforms are mutated [3]. The effects of mutations on p53α activities are well studied and include loss- and gain-of-functions [4], but their effect on p53 isoforms activities remains unknown. Several clinical studies demonstrated that the prognosis accuracy of cancer patients could be improved by combining *TP53* mutation status and p53 isoforms expression [2, 5–7]. Therefore, exploring the effect of mutations on p53 isoforms activities is a critical, albeit unexplored area in the p53 field.

Δ133p53α, one of the best characterized isoforms, is a negative regulator of senescence, particularly in normal cells [8–11]. Δ133p53α is also involved in DNA double-strand break repair and pluripotent stem cells regulation where it prevents genomic instability [12]. Importantly, wild-type (WT) Δ133p53α is non-oncogenic and non-mutagenic in normal human cells [12].

Glioblastoma (GBM) are the most aggressive brain tumors with only 6.8% five-year relative survival [13]. Current treatments, including temozolomide chemotherapy and radiations, induce cellular senescence through a p53-dependent mechanism [14, 15]. The progression from low-grade astrocytoma to glioblastoma is accompanied by *TP53* mutations [16]. Since Δ133p53α prevents cellular senescence, studying the impact of its mutation in glioblastoma is relevant to determine if it affects the progression and response to treatment of GBM cells and mutant *TP53* cancers in general, whether it may be used as a prognostic marker, and represent a potential therapeutic target.

Here, we identified a new mutant Δ133p53α-specific pathway in which it increases IDO1 (indoleamine-2,3-dioxygenase 1) and IL4I1 (interleukin-4 induced 1) expression and activates AHR (aryl hydrocarbon receptor), a tumor-promoting mechanism. We confirmed that mutant Δ133p53α R273H increases glioblastoma cells proliferation and invasion and prevents apoptosis while failing to maintain DNA stability. In addition, it also affects treatment response as WT and mutant Δ133p53α respond differently to temozolomide and radiation treatments.

## Material and methods

### Cell culture and treatments

Wild-type *TP53* (U87-MG, A172) and *TP53*-null (LNZ-308) came from ATCC. Mutant *TP53* cells (SF-268, SNB-19) came from NCI-Frederick cancer DCTD tumor/cell line repository. Cells were sequenced by CD Genomics [17] to confirm their IDH status and classification as GBM [18] and validate their *TP53* status. Cells were cultured at 37 °C, 5% CO2 in DMEM (Gibco®, Invitrogen) with 10% FBS, 1% l-Glutamine and 1% penicillin/streptomycin (Invitrogen). For 3D culture, cells were seeded in ultra-low attachment round bottom wells (S-bio) with Tumorsphere MediumXF (PromoCell) and incubated 4 days for spheroids formation before performing experiments.

When specified, cells were exposed to 50 μM temozolomide (TMZ, Sigma-Aldrich) for 5 days or irradiated with 10 Gy (X-Rad320 biologic irradiator, Precision X-ray) and analyzed 5 days later.

### Lentiviral transduction

Lentiviral transduction was performed as previously reported [8] and is detailed in supplementary methods.

### Western blot

Western blot procedure is detailed in supplementary methods. Antibodies used are listed in table S1 and full-size images in Fig. S5. MAP4, SAPU and KJC12 may be requested to the source mentioned in table S1 and were first published in [8, 19, 20], respectively.

### Transcriptome analysis

mRNAs were extracted using a Qiagen Rneasy-Plus kit according to the manufacturer’s instructions. mRNA sequencing (40 M reads, paired-end 150 bp) was performed on NovaSeq6000 (Illumina) by CCR sequencing facility (Frederick, MD, USA). Library preparation and reads alignment are detailed in supplementary methods. Analysis was performed using the NIH Integrated Data Analysis Platform [21] (NIDAP). Cutoff: Adjpval<0.01, |logFC| >1. GEO accession number GSE240377.

### Quantitative real-time PCR

qRT-PCR was performed as previously described [22]. Primers used (ThermoFisher) are listed in Table S2. The expression level was analyzed with the ΔΔCt method and normalized to GAPDH.

### Transfections

10 nM si133 (5′-GGAGGUGCUUACACAUGUU-3′) and siScramble (12,935-100) were transfected using Lipofectamine RNAiMAX (all Invitrogen) while WT and mutant p53α or pcDNA3 control plasmids (0.5 μg) were transfected using TurboFect (ThermoFisher Scientific) following manufacturer’s reverse-transfection protocol.

### Immunofluorescence staining

Immunofluorescence was performed as previously described [23]. Slides were mounted with Vectashield® Antifade mounting medium with DAPI (Vector laboratories) and imaged using Zeiss-780 confocal microscope. Antibodies used are listed in Table S1.

### Cell confluence and annexin-V live cell imaging

The growth medium was supplemented 1/400 with Incucyte® Annexin-V Dye (Sartorius). Phase (confluency) and GFP (Annexin-V) pictures were taken every 4 h by Incucyte-S3® (10x magnification).

### Homologous recombination assay

Cells were reverse-transfected with 0.18 μg/1.9cm^2^ pSCE and 0.36 μg/1.9cm^2^ DR-HRGFP plasmids from HR DNA reporter (TopoGEN). The next day, cells were treated with DMSO or TMZ and imaged 24 h later with Incucyte-S3® (10x magnification).

### Sulforhodamine-B (SRB) staining

Cells were fixed with ice-cold 100% methanol for 15 min and washed with water. Cells were incubated 30 min with SRB solution (0.4% SRB, 1% acetic acid) and washed with 1% acetic acid. SRB staining was solubilized using 10 mM unbuffered Tris and absorbance read at 570 nm.

### Transwell assay

Cells were serum-starved for 48 h and seeded in serum-free medium in Corning® Transwell® top chamber (8.0μm pore, Sigma-Aldrich). The bottom chamber contained 10% FBS medium. After 16 h, non-migrated and migrated cells were collected and counted.

### 3D cultures growth and invasion

Spheroids growth was measured every 4 h by Incucyte-S3® (10× magnification).

For invasion experiments, Tumorsphere MediumXF was removed and replaced by 50 μl Matrigel® (Corning). The plate was incubated 2 h at 37 °C for Matrigel® polymerization before adding 50 μl DMEM + 10% FCS as chemoattractant. Total area and invasion were measured every 4 h with Incucyte-S3® (4x magnification).

### IL-6 ELISA

Human IL-6 ELISA kit (Sigma-Aldrich) was used following the manufacturer’s instructions.

### Senescence assays

Senescence-associated-β-Galactosidase Staining Kit (CellSignaling Technology, #9860 S) was used following the manufacturer’s protocol.

CellEventTM Senescence Green Detection Kit (ThermoFisher) probe was diluted 1/2500 with buffer and incubated on cells 1 h at 37 °C. GFP signal was quantified with Incucyte-S3® (10x magnification).

### Statistical analysis

Data are presented as mean and standard deviation with comparisons made using a two-sided, unpaired Student’s *t* test, of at least three independent experiments. Differences were considered significant at a value of *p ≤ 0.05, **p ≤ 0.01, and ***p ≤ 0.001 or ns (not significant).

### TCGA RNA-sequencing analysis

IL4I1, IDO1, and AHR (FPKM) expression and IDH mutation data (to stratify cases based on the 2021 WHO directions^18^) from 669 primary glioma patients (516 LGG/ 153 GBM) from The Cancer Genome Atlas database were downloaded from cBioPortal [24]. Associated clinical information was obtained from Genomics Data Commons. GraphPad Prism was used to generate Kaplan–Meier overall-survival analysis and log-rank test to compare overall survival between groups with high (greater than the median) and low (lower than the median) IL4I1, IDO1, or AHR expression.

### Ethics statement and consent to participate

All patients’ data were downloaded from The Cancer Genome Atlas (TCGA), a public database, therefore no ethical approval was needed.

## Results

### Mutant Δ133p53α R273H induces IL4I1 and IDO1 expression and activates AHR

**Fig. 1:**
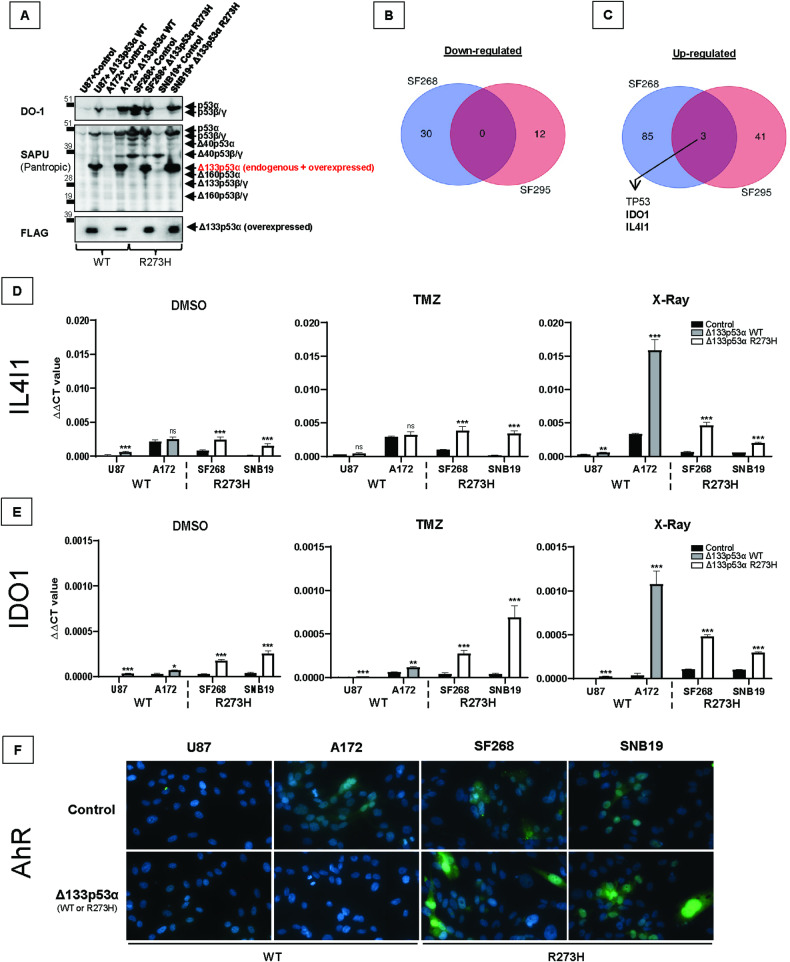
Mutant Δ133p53α R273H induces the IDO1/IL4I1/AHR pathway. **A** Western blot of the WT or mutant FLAG-Δ133p53α and endogenous p53 isoforms using DO-1 (full-length p53 isoforms) SAPU (p53 pantropic) or anti-FLAG antibodies. *n* = 3. **B**, **C** Venn diagrams representing the down-regulated and up-regulated genes in SF268 and SNB19 following mutant Δ133p53α R273H overexpression. *TP53* is logically found up-regulated since we overexpressed the Δ133p53α isoform. *n* = 3. **D**, **E** IL4I1 and IDO1 mRNA expression measured by Taqman in cells treated with DMSO (control), TMZ (50 μM for 5 days), or X-rays (10 Gy). ΔΔCt values were normalized to GAPDH. *n* = 3. **F** Immunofluorescence staining (40x magnification) of AHR (Green) and nuclear stain DAPI (blue). *n* = 3. AHR nuclear translocation is induced by mutant Δ133p53α R273H.

To study whether Δ133p53α mutation impacts its activities, we selected the R273H mutation since this is the most frequent mutation in GBM (13.5–11.8%) and the first or second most frequent mutation in cancers (6.4–5.9%) [3, 25]. Since Δ133p53α knock-down is not possible without also knocking-down Δ133p53β/γ and Δ160p53α/β/γ, we stably overexpressed WT Δ133p53α in WT *TP53* GBM cells (U87/A172), and mutant Δ133p53α R273H in R273H mutant *TP53* GBM cells (SF268/SNB19). We confirmed that both WT and mutant Δ133p53α are similarly overexpressed in nearly all cells using western blot (Fig. 1A) and immunofluorescence staining (Fig. S1A).

We treated Δ133p53α-expressing cells with temozolomide (TMZ) or X-rays and performed mRNA sequencing to determine the pathways and functions impacted by Δ133p53α mutation (Fig. S1B–I). In WT cells, we could not identify common target genes up- or down-regulated by WT Δ133p53α overexpression. Similarly, no mutually down-regulated genes could be identified in mutant Δ133p53α R273H-overexpressing cells (Fig. 1B).

Nevertheless, we identified that mutant Δ133p53α R273H upregulates two target genes, IDO1 and IL4I1, in both SF268 and SNB19 cells (Fig. 1C). Using qRT-PCR, we verified that IDO1 and IL4I1 are up-regulated by mutant Δ133p53α R273H overexpression (Fig. 1D, E) while their expression was not or minimally increased by WT Δ133p53α. Of note, they were highly increased by WT Δ133p53α in A172 cells, but not in U87 cells, and in response to radiations specifically, although these cell line- and treatment-specific changes are of unknown origin. To confirm that endogenous mutant Δ133p53α R273H also regulate IDO1 and IL4I1, we used si133 to knock-down the Δ133/Δ160 transcripts, including the Δ133p53α-encoding transcript [2] (Genbank NM_001126115), along with or without overexpression, which resulted in IDO1 and IL4I1 down-regulation (Fig. S1J–L).

IDO1 and IL4I1 are involved in a common pathway where they induce AHR activation and nuclear translocation [26, 27]. Basal AHR expression is higher and more nuclear in mutant versus WT cells (Fig. 1F). Importantly, AHR is induced and translocated to the nucleus only upon mutant Δ133p53α R273H overexpression. Not all mutant Δ133p53α R273H-overexpressing cells are AHR positive, which might be explained by mutant Δ133p53α R273H heterogenous expression within cell population (Fig. S1A). Furthermore, the IL4I1/IDO1/AHR axis promotes tumor progression and aggressiveness [26–30] which is consistent with our Gene Set Enrichment Analysis (GSEA) indicating that mutant Δ133p53α R273H overexpression correlates with tumorigenic hallmarks (Fig. S1M). These results indicate that mutant Δ133p53α R273H may have acquired oncogenic activities.

### Mutant Δ133p53α R273H lost DNA repair but retains anti-apoptotic functions

**Fig. 2:**
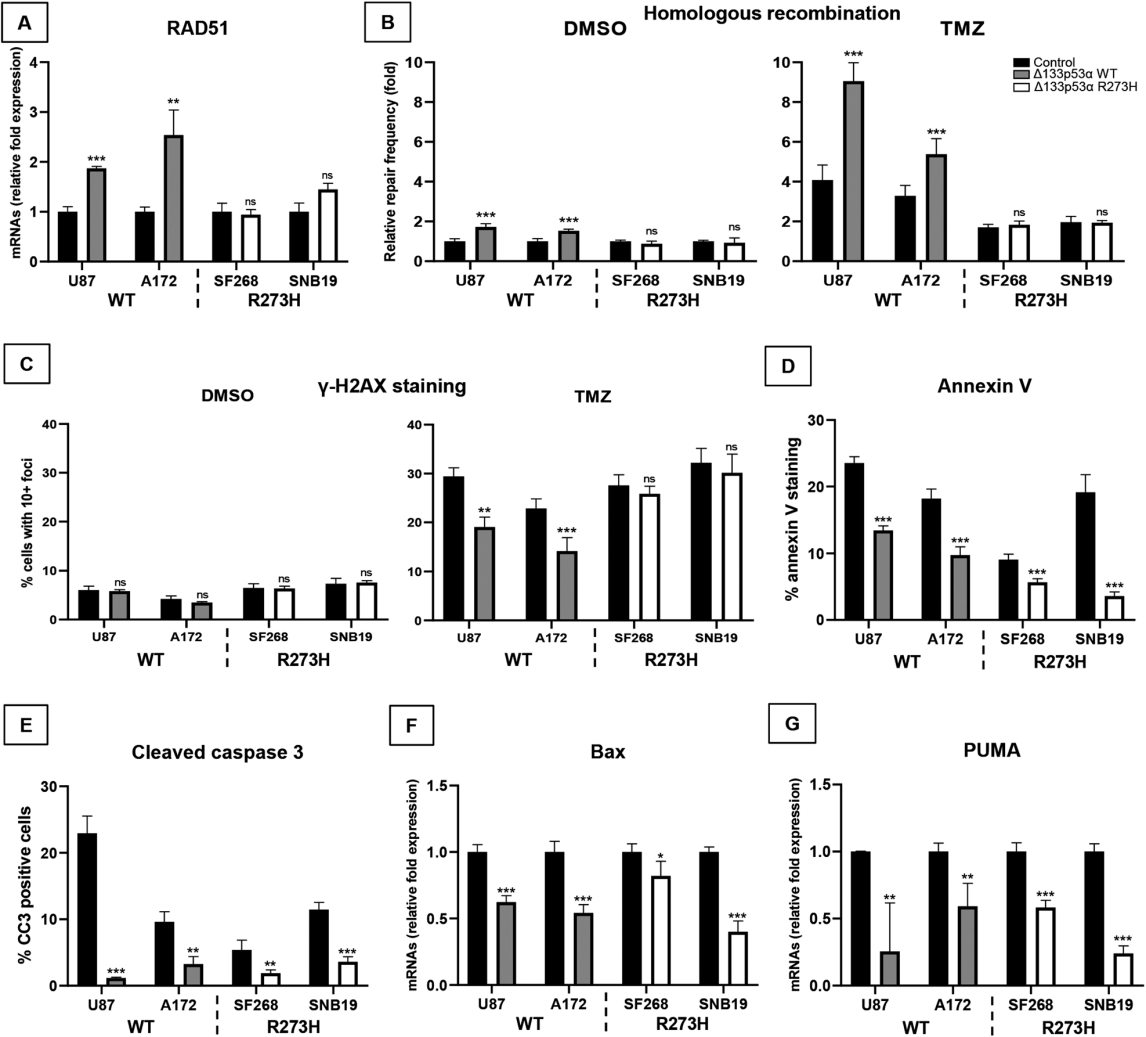
Mutant Δ133p53α R273H fails to promote DNA repair but retains anti-apoptotic functions. **A** Fold change of RAD51 mRNA expression measured by Taqman. *n* = 3. **B** The frequency of homologous recombination was measured on cells treated with DMSO (control) or TMZ (50 μM) 24 h after treatment beginning and expressed as fold change of the control-DMSO sample for each cell line. *n* = 3. **C** γ-H2AX staining was performed on cells treated with DMSO (control) or TMZ (50 μM for 5 days). The percentage of cells with at least 10 foci was determined (corresponding images in Fig. S2B). *n* = 3. **D** Cells were grown in the presence of annexin-V dye in Incucyte® and the percentage of annexin-V staining was determined after 80 h. *n* = 4. **E** Quantification of the percentage of cells positive for Cleaved Caspases 3 by immunofluorescence (corresponding images in Fig. S2C). *n* = 3. **F**, **G** Bax and PUMA mRNA expression was measured by Taqman. *n* = 3.

Δ133p53α promotes DNA repair through DNA repair genes upregulation, including LIG4, RAD51, and RAD52 [31, 32]. Hence, Δ133p53α maintains DNA integrity in induced pluripotent stem cells and enhances DNA repair in prematurely aged cells through p53α inhibition and E2F1 activation [33–35]. Using qRT-PCR, we verified that WT Δ133p53α overexpression increases RAD51 expression while mutant Δ133p53α R273H did not affect it (Fig. 2A). Using Δ133 isoforms siRNA knock-down, we confirmed that, contrarily to the WT protein, endogenous mutant Δ133p53α R273H does not contribute to RAD51 expression (Fig. S2A), suggesting that it may have lost its DNA repair function.

As RAD51 is a major effector of homologous recombination [36], we quantified HR repair following DMSO or Temozolomide treatment. TMZ, an alkylating agent, induces DNA damage and represses E2F1-associated DNA repair pathway [14], the pathway Δ133p53α activates to promote DNA repair [33–35]. Consistently, WT Δ133p53α induced HR in both control and TMZ treated cells while the mutant did not have any effect (Fig. 2B).

Next, we performed γ-H2AX staining to quantify DNA double-strand breaks. Under control conditions, the low DNA-damaged cells percentage did not allow significant differences detection (Fig. 2C, S2B). Following TMZ treatment, however, WT Δ133p53α-overexpressing cells harbored a reduced number of γ-H2AX foci while mutant Δ133p53α R273H had no effect, confirming that the mutation impaired Δ133p53α DNA repair activity.

To favor DNA repair, WT Δ133p53α represses p53α-mediated apoptosis [8, 37]. Since the IL4I1/IDO1/AHR axis prevents apoptosis [30, 38], mutant Δ133p53α R273H may retain its anti-apoptotic functions. Performing annexin-V and cleaved caspase-3 staining, we found that both WT and mutant Δ133p53α reduced spontaneous apoptosis (Fig. 2D, E, S2C). Using si133, we confirmed that both WT and mutant Δ133p53α negatively regulate apoptosis at the endogenous level (Fig. S2D). Furthermore, we also performed annexin-V staining in p53-null LNZ-308 cells and neither WT nor mutant Δ133p53α reduced annexin-V staining (Figs. S2E, F). This is consistent with WT Δ133p53α repressing p53α-mediated apoptosis [8, 37] and indicates that Δ133p53α R273H may also reduce apoptosis in a p53-dependent manner.

To investigate how Δ133p53α represses apoptosis, we quantified Bax and PUMA expression in Δ133p53α-expressing cells. Both genes were reduced by WT and mutant Δ133p53α overexpression (Fig. 2F, G). Consistently, Bax and PUMA were up-regulated upon endogenous Δ133p53 isoforms knock-down (Figs. S2G, H), confirming the negative correlation between Δ133p53α (WT or R273H) and Bax/PUMA expression. Altogether, these results confirm that WT Δ133p53α inhibits apoptosis to favor DNA repair but does not prevent p53α-dependent apoptosis in severely damaged cells [33, 34]. However, mutant Δ133p53α R273H has lost DNA repair capabilities and, therefore, cannot maintain genetic stability while blocking damaged cell elimination, demonstrating that it acquired oncogenic functions.

### Mutant Δ133p53α R273H promotes cell growth and invasion

**Fig. 3:**
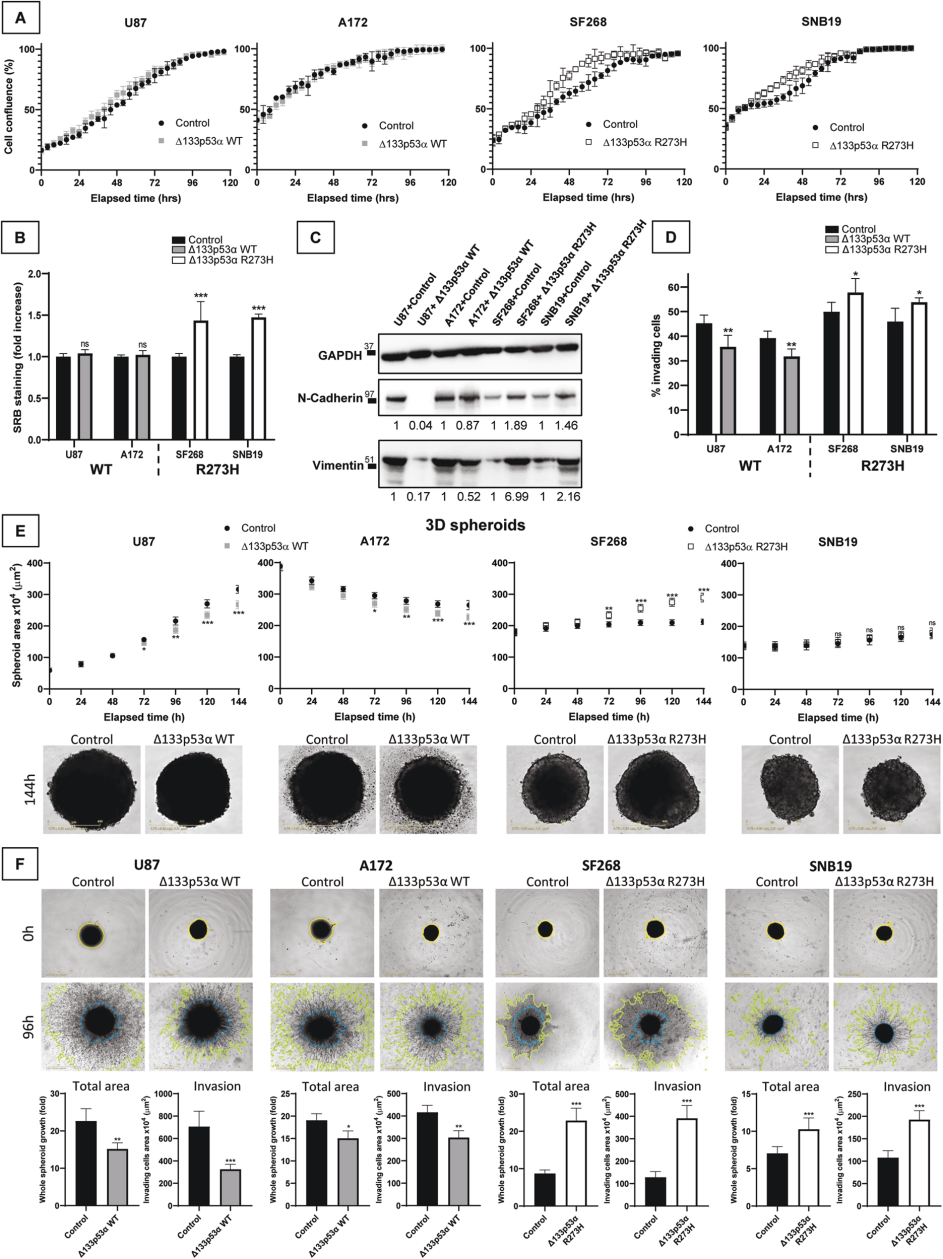
Mutant Δ133p53α R273H increases cell growth and cellular invasion. **A** Cells were imaged every 4 h in Incucyte® over 5 days and the percentage of confluence was measured. *n* = 4. **B** Cells were seeded at low density and left growing for 6 days before SRB staining was performed to determine cell growth. *n* = 8. **C** Protein expression of N-cadherin and Vimentin by Western blot. GAPDH was used as a loading control. *n* = 3. **D** Transwell assay was used to determine the percentage of invading cells 16 h after seeding. *n* = 5. **E** Cell spheroids were imaged every 4 h in Incucyte® over 6 days and the spheroid surface was measured. *n* = 4. **F** Spheroids were embedded in Matrigel and imaged every 4 h in Incucyte® over 4 days. The total spheroid and invasion area were measured. In the pictures, the yellow or blue line shows the spheroid core size, while the green line shows the limits of the spheroid total size/invasion. Invasion thus corresponds to the surface between the blue and the green lines. *n* = 8.

Previous studies of IL4I1/IDO1/AHR axis and our GSEA data both suggest that mutant Δ133p53α R273H may promote cell proliferation [26, 27, 30] (Fig. S1M). Our results showed that WT Δ133p53α-overexpressing cells grew similarly to control cells, while mutant Δ133p53α R273H expression minimally increased cell proliferation (Fig. 3A). This was reproducible in p53-null LNZ308 cells (Fig. S3A), suggesting that mutant Δ133p53α R273H may promote cell growth in a p53α-independent manner, potentially via the IL4I1/IDO1/AHR axis. Using sulforhodamine-B (SRB) staining, we confirmed that WT Δ133p53α did not affect the cellular growth rate, while mutant Δ133p53α increased proliferation by approximately forty-five percent (Fig. 3B). Furthermore, WT Δ133p53α knock-down did not affect cell growth while mutant Δ133p53α R273H depletion decreased cell proliferation, including in the Δ133p53α R273H-overexpressing cells (Fig. S3B).

Both the IL4I1/IDO1/AHR literature and our GSEA indicate that mutant Δ133p53α may increase EMT and cellular invasion [26, 27, 29] (Fig. S1M). Hence, mutant Δ133p53α R273H induced both N-cadherin and vimentin expression, while WT Δ133p53α reduced it (Fig. 3C). Using transwell assay, we observed that WT Δ133p53α overexpression slightly reduced cell invasion (Fig. 3D) while mutant Δ133p53α R273H overexpression significantly increased the percentage of invading cells, further suggesting mutant Δ133p53α oncogenic potential. In p53-null LNZ308 cells, WT Δ133p53α did not affect cell invasion, suggesting a p53-mediated function, while mutant Δ133p53α R273H still increased invasion, indicating a p53-independent mechanism, potentially through the IL4I1/IDO1/AHR axis (Fig. S3C).

To verify our results in more physiologically relevant models, we performed 3D spheroids/organoids culture which exhibit structural, morphogenetic, and functional properties that recapitulate In Vivo physiopathology [39, 40]. We first performed spheroids formation and growth assays (Fig. 3E) and determined that WT Δ133p53α significantly reduced spheroids growth compared to control. On the contrary, mutant Δ133p53α R273H increased spheroid growth in both mutant cell lines although significantly only in SF268 cells. This is consistent with our 2D results and confirms the increased capacity of mutant Δ133p53α R273H expressing cells to grow better and faster as tumorspheres.

Next, we embedded the spheroids in Matrigel to study their growth and invasion when interacting with an extra-cellular matrix (Fig. 3F). In this context, WT Δ133p53α significantly reduced tumorspheres growth and invasion while mutant Δ133p53α R273H increased both, consistently with our 2D results and confirming that mutant Δ133p53α R273H acquired oncogenic activities even when grown as organoids within an extra-cellular matrix.

### R273H mutation alters Δ133p53α regulation of cellular senescence, particularly in response to treatment

**Fig. 4:**
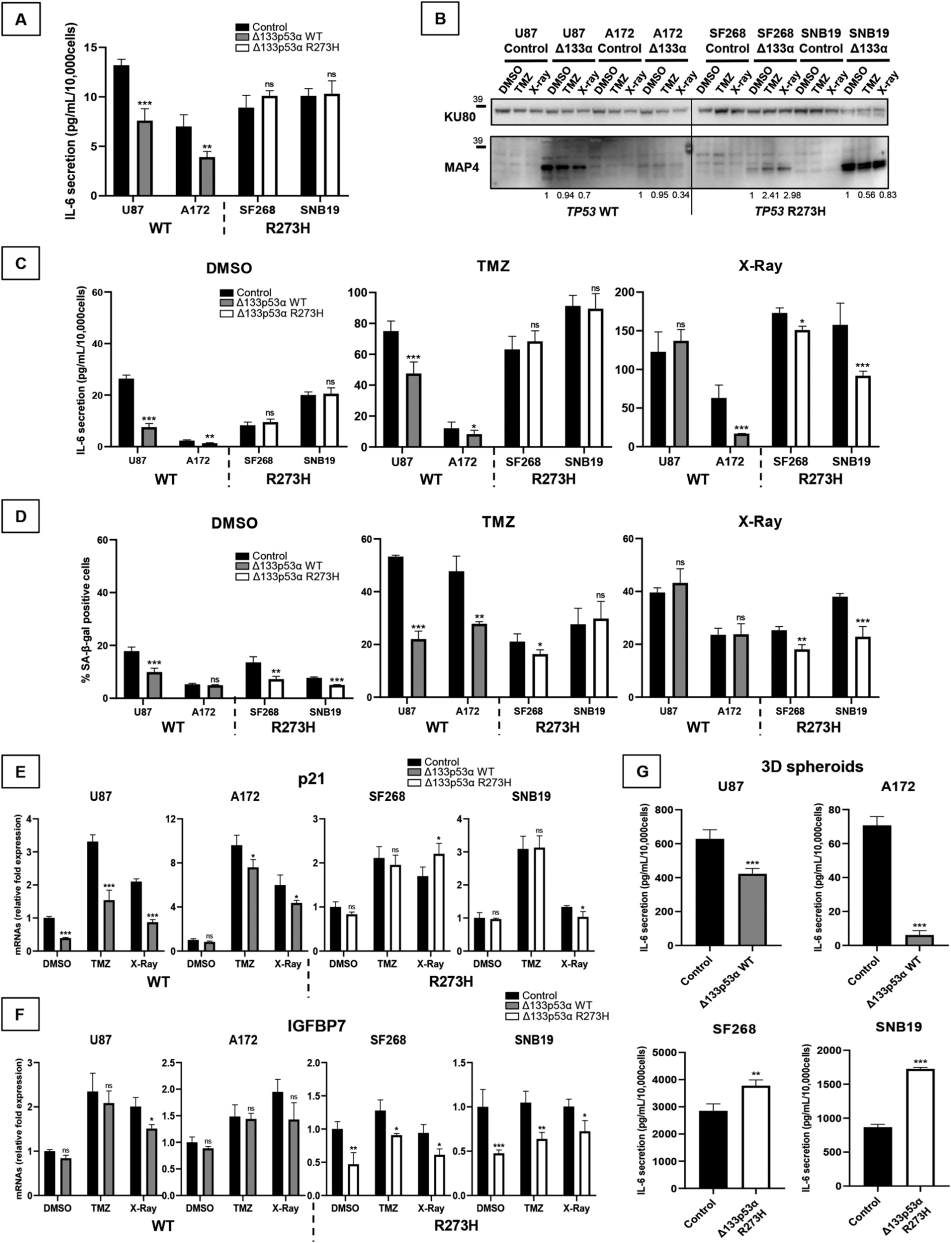
R273H mutation alters Δ133p53α regulation of cellular senescence, particularly in response to treatment. **A** IL-6 secreted in growth media was measured by ELISA after 5 days. IL-6 secretion was normalized to the number of cells. *n* = 4. **B**–**F** Cells were treated with DMSO (control), TMZ (50 μM for 5 days), or X-rays (10 Gy). **B** Western blot of Δ133p53 isoforms (MAP4 antibody) was performed and KU80 was used as a loading control. *n* = 3. **C** IL-6 secretion was measured by ELISA and normalized to cell number. *n* = 3. **D** The percentage of senescent cells was determined by Senescence-associated β-galactosidase staining. *n* = 3. **E**, **F** p21 and IGFBP7 mRNA expression was measured by Taqman. *n* = 3. **G** The IL-6 secreted in growth media by the spheroids was measured by ELISA after 7 days. IL-6 secretion was normalized to the number of cells. *n* = 3.

Our transcriptome analysis suggested that mutant Δ133p53α R273H induces inflammation via IL-6/JAK/STAT3, IL-2/STAT5, and TNFα/NFκB pathways (Fig. S1M) while WT Δ133p53α reduces cellular senescence through inhibition of the senescence-associated secretory phenotype (SASP), including many inflammation factors [8–10, 23, 33]. To investigate whether R273H mutation alters Δ133p53α regulation of senescence and SASP, we first measured IL-6 secretion. Consistent with previous findings [8–10, 23, 33], WT Δ133p53α overexpression reduced IL-6 secretion (Fig. 4A). In contrast, mutant Δ133p53α R273H did not affect IL-6 secretion. Furthermore, following Δ133p53 isoforms knock-down, IL-6 secretion was rescued in the WT cells but was unchanged in the mutant cells (Fig. S4A).

TMZ and radiation treatments are known cellular senescence inducers through a p53-dependent mechanism [14, 15]. Both treatments reduced WT Δ133p53α expression, with radiation causing the greatest effect (Fig. 4B). While differences appeared between the mutant cells, in SNB19, mutant Δ133p53α R273H was reduced by TMZ, but not by radiation, suggesting that mutant and WT cells may respond differently to treatments. Both treatments induced IL-6 secretion in WT and mutant cells (Fig. 4C). In U87 cells, WT Δ133p53α overexpression lowered IL-6 secretion in response to TMZ only. In A172 cells, IL-6 secretion was also decreased upon radiations which could be linked to the IDO1 and IL4I1 increase observed in response to radiations only in A172 cells (Fig. 1D, E). This indicates that WT Δ133p53α consistently counteracts TMZ-induced, but not radiation-induced IL-6 secretion. In contrast, mutant Δ133p53α R273H only decreased radiation-induced IL-6 secretion (Fig. 4C).

In the p53-null LNZ-308 cells, neither WT nor mutant Δ133p53α overexpression affected IL-6 secretion (Fig. S4B), probably because Δ133p53α represses IL-6 secretion mainly through interaction with canonical p53α [8–10, 23, 33]. To test this hypothesis, we transiently overexpressed p53α in our GBM cells. Both WT and mutant p53α increased IL-6 secretion, consistent with previous reports that p53α R273H up-regulates pro-inflammatory pathways [41] (Fig. S4C, D). When co-overexpressed, only WT Δ133p53α counteracted WT p53α effect, while mutant Δ133p53α R273H could not prevent p53α R273H induction of IL-6 secretion. These results confirm WT Δ133p53α p53α-dependent mechanism while mutant Δ133p53α lost this function.

Next, we asked whether IL-6 secretion correlated with cellular senescence. Both WT and mutant Δ133p53α reduced cellular senescence under control conditions (Fig. 4D). WT Δ133p53α rescued TMZ-induced senescence, but not radiation-induced senescence, while mutant Δ133p53α R273H rescued radiation-induced senescence but not TMZ-induced senescence, which is consistent with the IL-6 secretion above (Fig. 4C). We confirmed these results using a quantifiable, GFP-based method which displayed the same regulation (Fig. S4E).

Although both can reduce senescence, WT and mutant Δ133p53α respond differently to treatment suggesting that they act through different mechanisms. We quantified senescence-associated target genes and found that WT Δ133p53α reduces p21 expression while mutant Δ133p53α R273H does not affect it (Fig. 4E). In contrast, mutant Δ133p53α R273H, but not the WT, reduced IGFBP7 expression (Fig. 4F). We confirmed that p21 expression was up-regulated by WT Δ133p53α knock-down only, while IGFBP7 was up-regulated upon mutant Δ133p53α depletion only (Figs. S4F, G). IGFBP7 downregulation was reported to escape p53α-induced senescence [42, 43] which indicates a switch from cell cycle arrest to senescence-associated IGF pathway induced by Δ133p53α mutation. The different target-gene selection may explain why both WT and mutant Δ133p53α can reduce cellular senescence in the absence of treatment, and their different response to treatment.

Lastly, we measured the level of IL-6 secreted by the spheroids (Fig. 4G). When 3D cultured, WT Δ133p53α consistently reduced IL-6 secretion while mutant Δ133p53α R273H significantly increased it. This shows that mutant Δ133p53α R273H increases IL-6 when evaluated in more physiologically relevant conditions. These data suggest that mutant Δ133p53α R273H may promote a highly inflammatory environment which is consistent with the upregulated inflammatory response and TNFα/NFκB pathway in mutant Δ133p53α R273H-overexpressing cells (Fig. S1M). This also supports the conclusion that mutant Δ133p53α R273H acquired tumorigenic activity since tumor-promoting inflammation is a hallmark of cancer [44].

### IL4I1 expression is upregulated in GBM and is associated with poorer survival in GBM and LGG cohorts

**Fig. 5:**
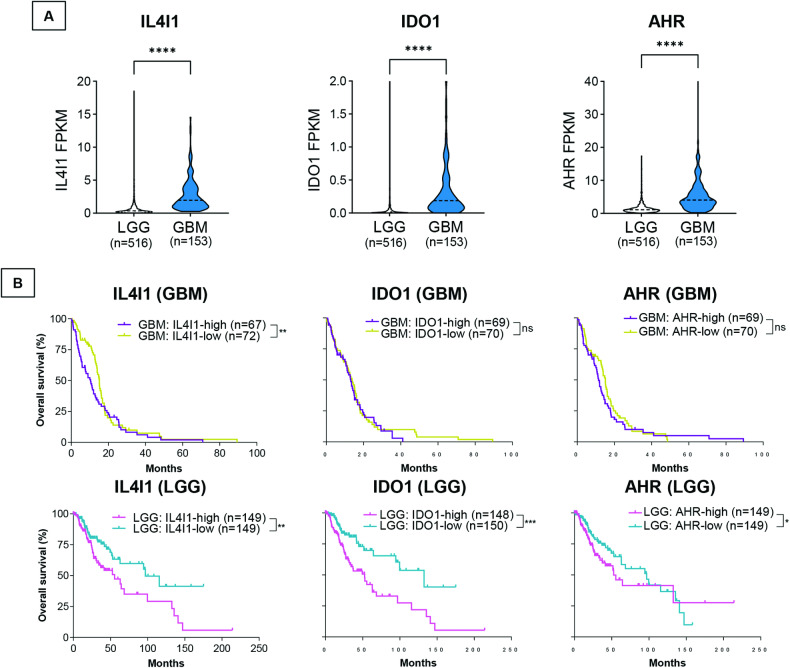
Expression of IL4I1, IDO1 and AHR is upregulated in glioblastoma patients compared to low-grade glioma patients and high levels of IL4I1 are significantly associated with *TP53* mutant tumors and poorer survival. **A** Expression of IL4I1, IDO1 and AHR in glioblastoma (GBM, *n* = 153) compared to low-grade glioma patients (LGG, *n* = 516). Mann–Whitney test was used to analyze statistical significance. **B** Kaplan–Meier curves depicting the overall survival of GBM or LGG patients expressing high (higher than median) or low (lower than median) levels of IL4I1, IDO1, and AHR. Log-rank Mantel-Cox was used to determine significance.

We examined IL4I1, IDO1, and AHR expression in the TCGA database and their contribution to glioblastoma and low-grade glioma (LGG) clinical outcome. We found that IL4I1, IDO1, and AHR expression are significantly higher in GBMs versus LGGs (Fig. 5A).

We next stratified GBM and LGG patients by high or low IL4I1, IDO1 or AHR expression. Notably, higher IL4I1 expression is associated with shorter survival of GBM and LGG patients (Fig. 5B), while higher IDO1 and AHR expression were associated with poorer survival of LGG patients only. These results are consistent with our In Vitro data showing that IL4I1/IDO1 upregulation and AHR activation by mutant Δ133p53α lead to higher tumor cell migration and invasion. Overall, these findings underscore IL4I1 prognostic significance in GBM and LGG and strengthen the potential therapeutic value of targeting IL4I1 expression in these tumors.

## Discussion

Here, we identified a novel, mutant-specific Δ133p53α/IDO1/IL4I1/AHR axis that promote tumor progression and aggressiveness, including reduced apoptosis, increased proliferation, and resistance to treatment [26–30, 38, 45–47]. We report for the first time that IL4I1, IDO1, and AHR are all three increased in GBM compared to LGG. Furthermore, we show that IL4I1, IDO1, or AHR high expression correlates with poorer survival of LGG patients, and that high IL4I1 expression also correlates with poorer outcome in GBM patients, confirming the clinical relevance of this pathway in these cancers.

**Fig. 6:**
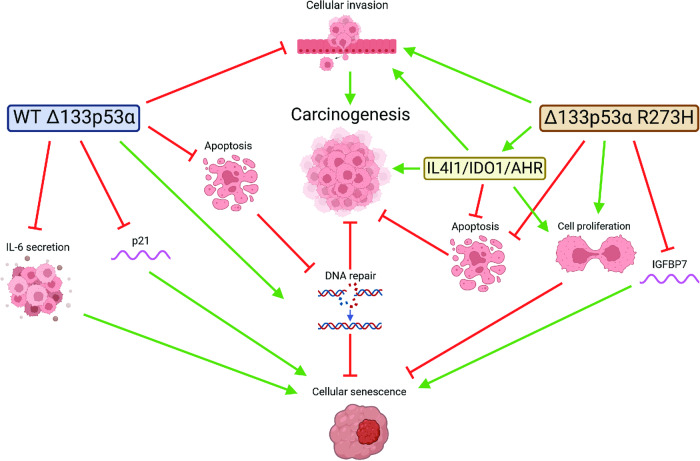
Model-mutant Δ133p53α R273H mutation reorientates its activities towards carcinogenesis. Created with BioRender.com.

Using 2D and 3D techniques, we determined that mutant Δ133p53α R273H increases proliferation, invasion, DNA instability, and inflammation while reducing apoptosis and cell cycle arrest genes in glioblastoma cells (Fig. 6). In addition to immunosuppression driven by IDO1/IL4I1/AHR axis upregulation [48, 49], our data demonstrate that, compared to the WT, mutant Δ133p53α R273H has acquired at least seven out of the ten hallmarks of cancers [44] and that it has gained oncogenic function. Lastly, we determined that Δ133p53α may have an impact on the patient’s response to treatment. Indeed, both WT and mutant Δ133p53α reduce cellular senescence and expression of senescence-associated genes, but in a treatment-dependent manner. Importantly, while apoptosis and senescence are p53-dependent, the increased cellular proliferation and invasion are mutant p53α-independent, indicating that these activities are intrinsic and actively carried by mutant Δ133p53α R273H, potentially through IL4I1/IDO1/AHR.

Altogether, these findings provide novel mechanistic insights into mutant Δ133p53α R273H activities and demonstrate that it is an active contributor to glioblastoma carcinogenesis and response to treatment. Hence, a mutant TP53 tumor may be more aggressive and not respond to treatment the same way if it expresses mutant Δ133p53α, underlying the importance of combining *TP53* mutation status and isoforms expression. By discovering the link between *TP53* mutation and IL4I1/IDO1/AHR pathway, we show strong evidence of Δ133p53α R273H clinical relevance in mutant *TP53* glioblastoma development and aggressiveness, and as a potential therapeutic target and biomarker. Indeed, targeting IL4I1, IDO1 or AHR in mutant *TP53* tumors may offer new clinical opportunities [50, 51]. Several IDO1 small-molecule inhibitors are in clinical trials for advanced melanoma [52, 53]. While the first phase III trial was not conclusive [54], specifically targeting mutant *TP53* tumors or combining it with mutant Δ133p53α-targeting drugs may improve effectiveness. Similarly, several AHR antagonists exist, and piperazine-2,3-dione derivatives were suggested as selective IL4I1 inhibitors [55]. Interestingly, IL4I1 is secreted and found in serum [56] where it promotes a tumor-prone microenvironment, increasing the concentration of metabolites in the patient’s biological fluids, including malignant gliomas patients’ cerebrospinal fluid [57–59]. Therefore, this may be an opportunity to detect mutant Δ133p53α R273H-induced expression of IL4I1 in mutant *TP53* tumors in a non-invasive and easier way .

### Supplementary information

Supplementary information

## Data Availability

Data generated for this manuscript will be made available upon reasonable request to the corresponding author. The mRNA sequencing results have been deposited to the Gene Expression Omnibus (GEO) and can be found under the accession number GSE240377.
