# Supplementary material for: Distinct functions of wild-type and R273H mutant Δ133p53α differentially regulate glioblastoma aggressiveness and therapy-induced senescence
Source: Cell Death Dis. 2024 Jun 27;15(6):454. doi: 10.1038/s41419-024-06769-5 (PMC11211456; doi:10.1038/s41419-024-06769-5)
Supplement: Supplementary file 1 — Supplementary information [file 41419_2024_6769_MOESM1_ESM.pdf]

Supplementary figures

Supplementary figure 1

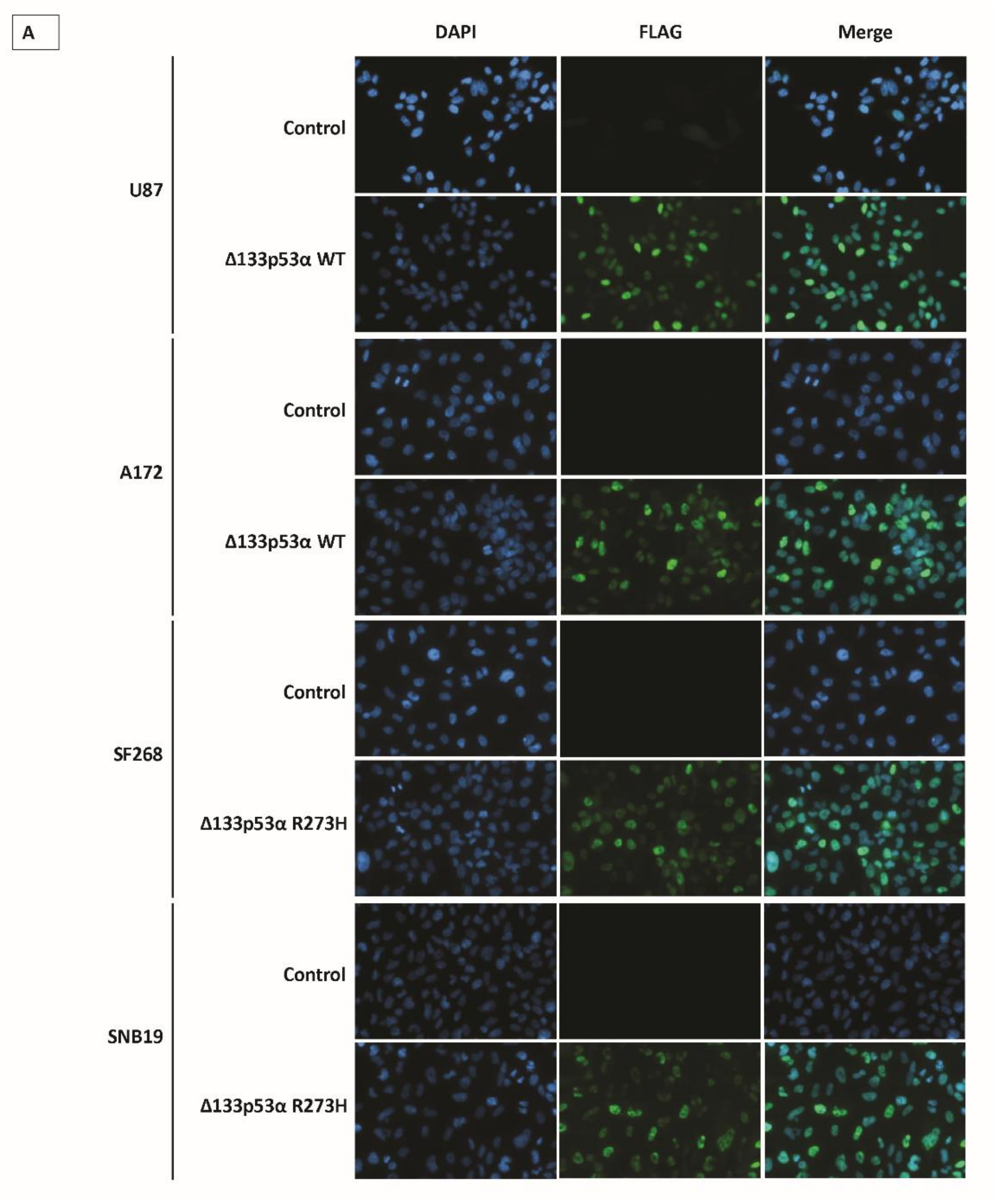

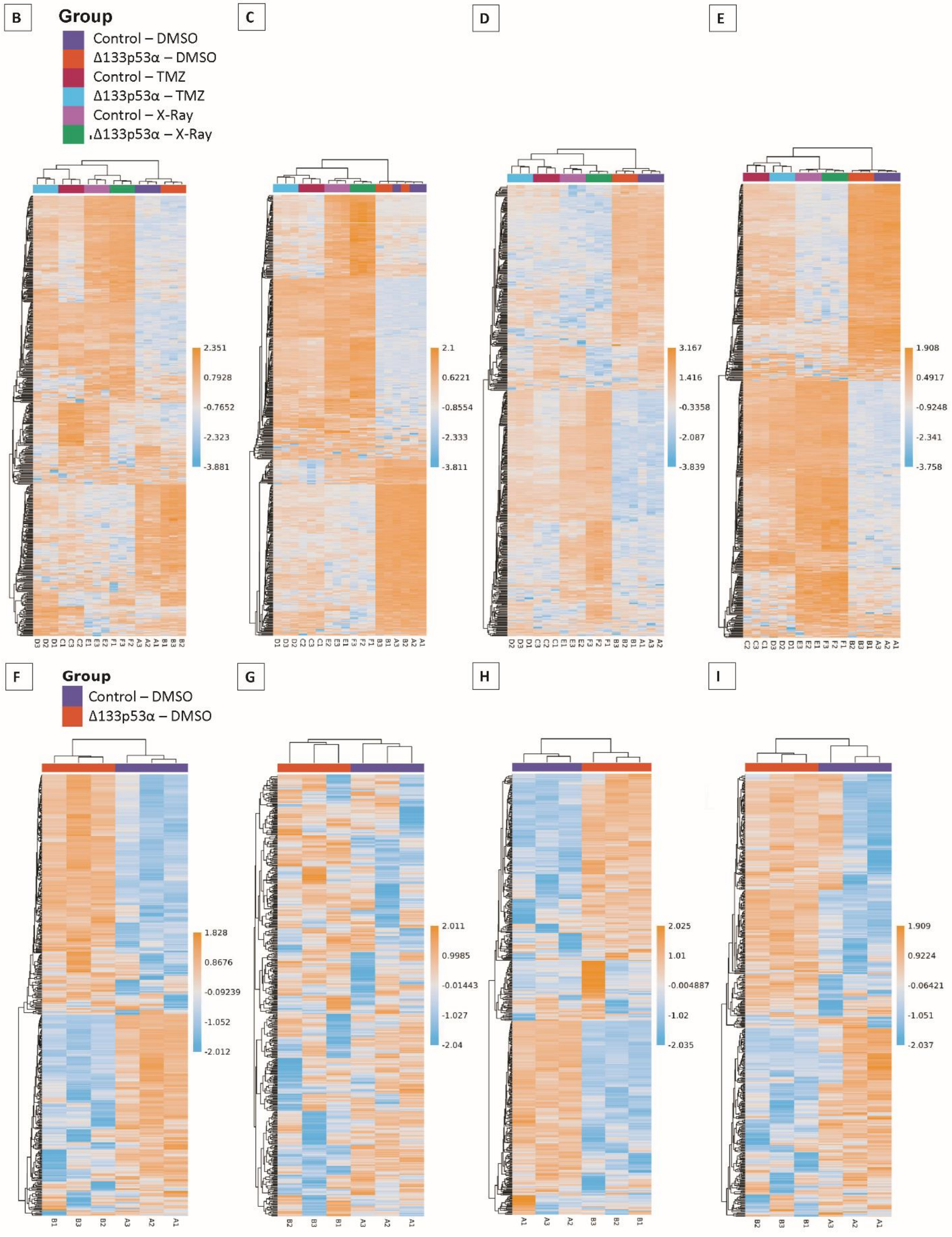
B
Group
Control – DMSO
Δ133p53α – DMSO
Control – TMZ
Δ133p53α – TMZ
Control – X-Ray
Δ133p53α – X-Ray
2.351
0.7928
-0.7652
-2.323
-3.881
C
2.1
0.6221
-0.8554
-2.333
-3.811
D
3.167
1.416
-0.3358
-2.087
-3.839
E
1.908
0.4917
-0.9248
-2.341
-3.758
F
Group
Control – DMSO
Δ133p53α – DMSO
1.828
0.8676
-0.09239
-1.052
-2.012
G
2.011
0.9985
-0.01443
-1.027
-2.04
H
2.025
1.01
-0.004887
-1.02
-2.035
I
1.909
0.9224
-0.06421
-1.051
-2.037

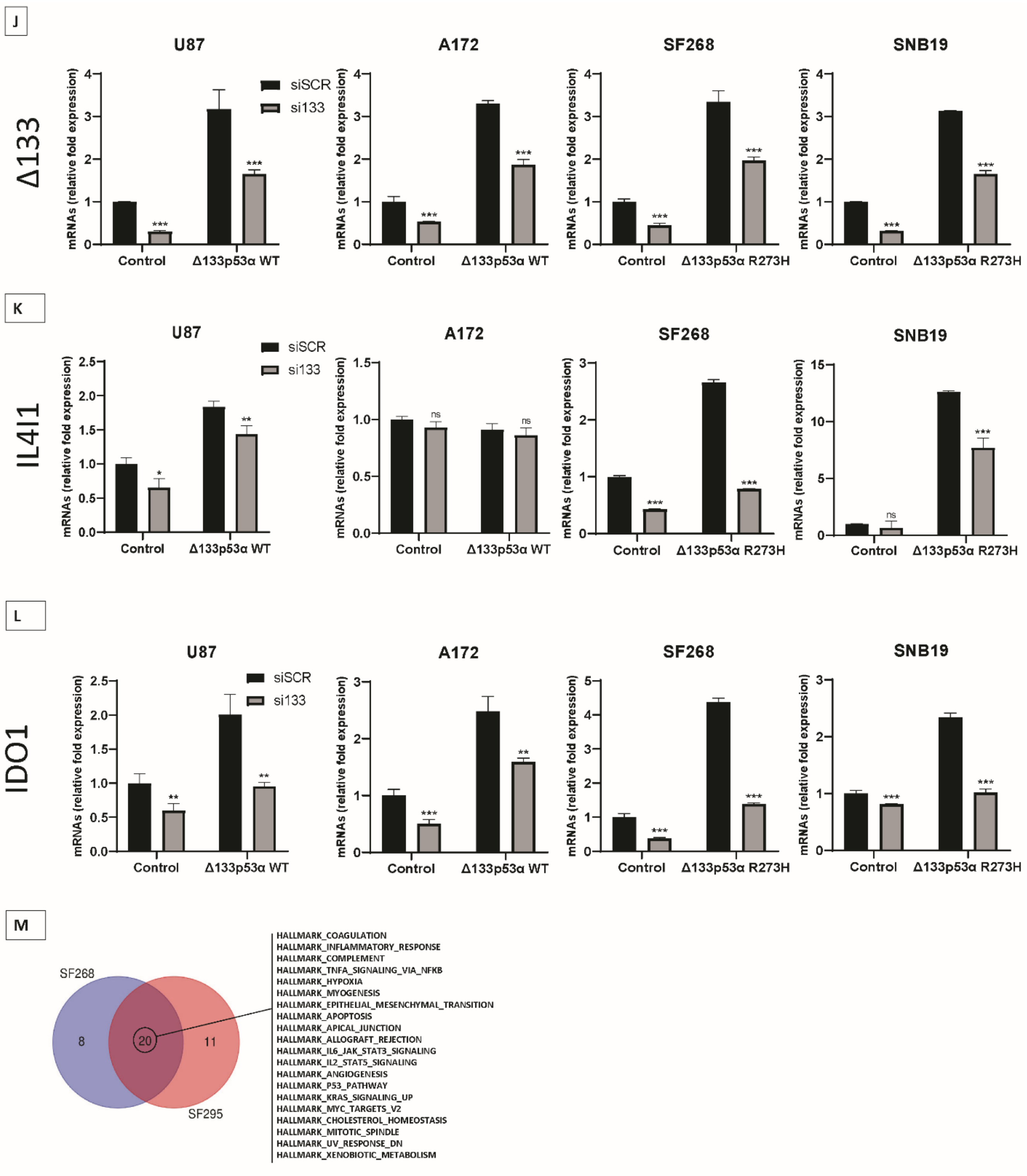
J
Δ133
U87
A172
SF268
SNB19
siSCR
si133
mRNAs (relative fold expression)
Control
Δ133p53α WT
Δ133p53α R273H
K
IL4I1
U87
A172
SF268
SNB19
siSCR
si133
mRNAs (relative fold expression)
Control
Δ133p53α WT
Δ133p53α R273H
L
IDO1
U87
A172
SF268
SNB19
siSCR
si133
mRNAs (relative fold expression)
Control
Δ133p53α WT
Δ133p53α R273H
M
SF268
SF295
8
20
11
HALLMARK_COAGULATION
HALLMARK_INFLAMMATORY_RESPONSE
HALLMARK_COMPLEMENT
HALLMARK_TNFA_SIGNALING_VIA_NFKB
HALLMARK_HYPOXIA
HALLMARK_MYOGENESIS
HALLMARK_EPITHELIAL_MESENCHYMAL_TRANSITION
HALLMARK_APOPTOSIS
HALLMARK_APICAL_JUNCTION
HALLMARK_ALLOGRAFT_REJECTION
HALLMARK_IL6_JAK_STAT3_SIGNALING
HALLMARK_IL2_STAT5_SIGNALING
HALLMARK_ANGIOGENESIS
HALLMARK_P53_PATHWAY
HALLMARK_KRAS_SIGNALING_UP
HALLMARK_MYC_TARGETS_V2
HALLMARK_CHOLESTEROL_HOMEOSTASIS
HALLMARK_MITOTIC_SPINDLE
HALLMARK_UV_RESPONSE_DN
HALLMARK_XENOBIOTIC_METABOLISM

Fig. S1: Mutant Δ133p53α R273H induces the IDO1/IL4I1/AHR pathway. **A)** Immunofluorescence staining (40x magnification) of overexpressed FLAG-Δ133p53α WT or R273H (Green) and nuclear stain DAPI (blue). n=3. **B to E)** Heatmap representation of genes up- and down regulated following WT or mutant Δ133p53α overexpression and DMSO (control), TMZ (50μM for 5 days), or X-rays (10Gy) treatment in U87 (B), A172 (C), SF268 (D), and SNB19 (E) cell lines. n=3. **F to I)** Heatmap representation of genes up- and down regulated following WT or mutant Δ133p53α overexpression in U87 (F), A172 (G), SF268 (H), and SNB19 (I) cell lines. n=3. **J to L)** Δ133 isoforms were knocked down by siRNA. Taqman was used to determine Δ133p53, IDO1, and IL4I1 mRNA expression respectively. n=3. **M)** Venn diagram representing the up-regulated pathways identified by our gene set enrichment analysis in SF268 and SNB19 cells.

Supplementary figure 2

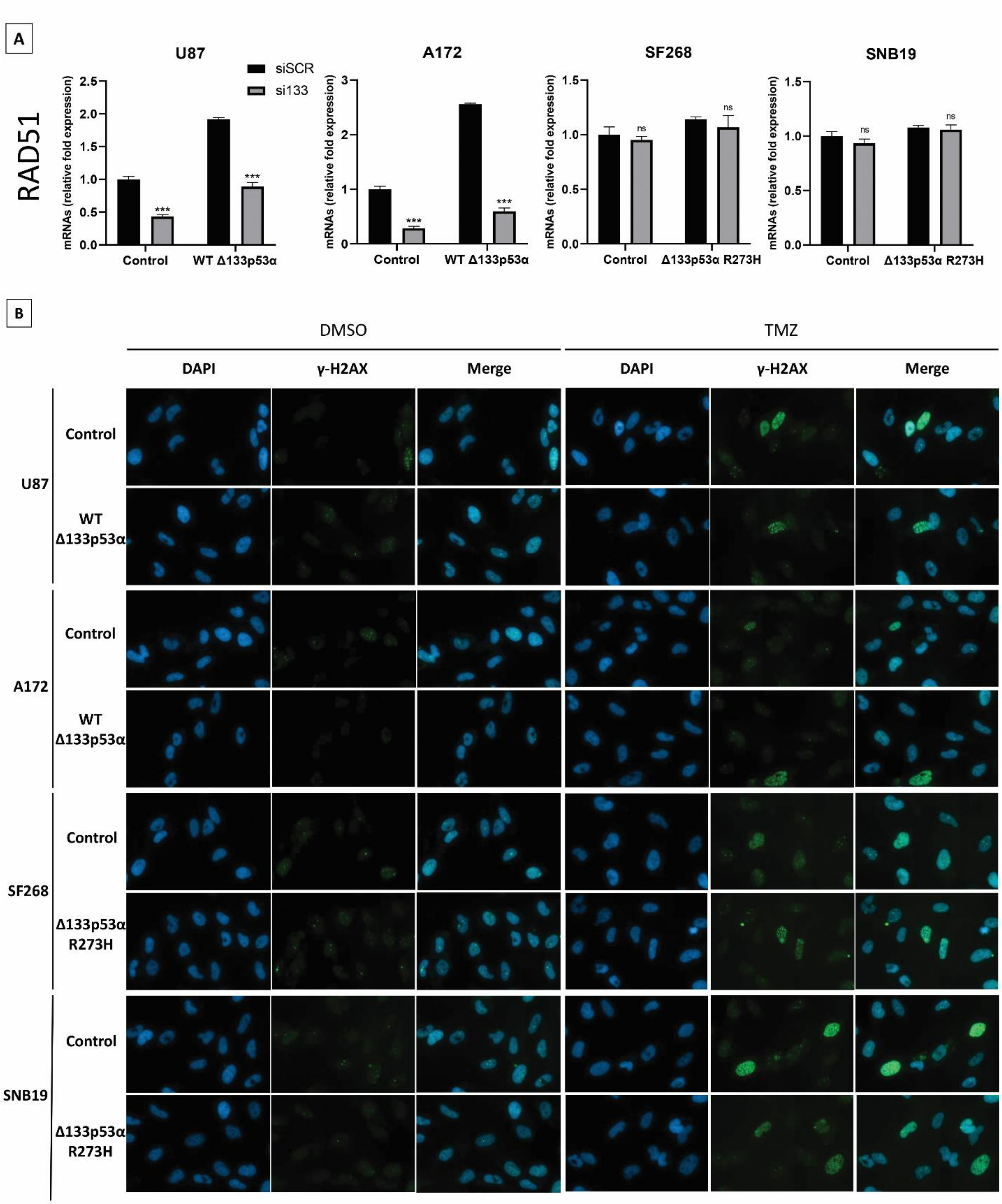

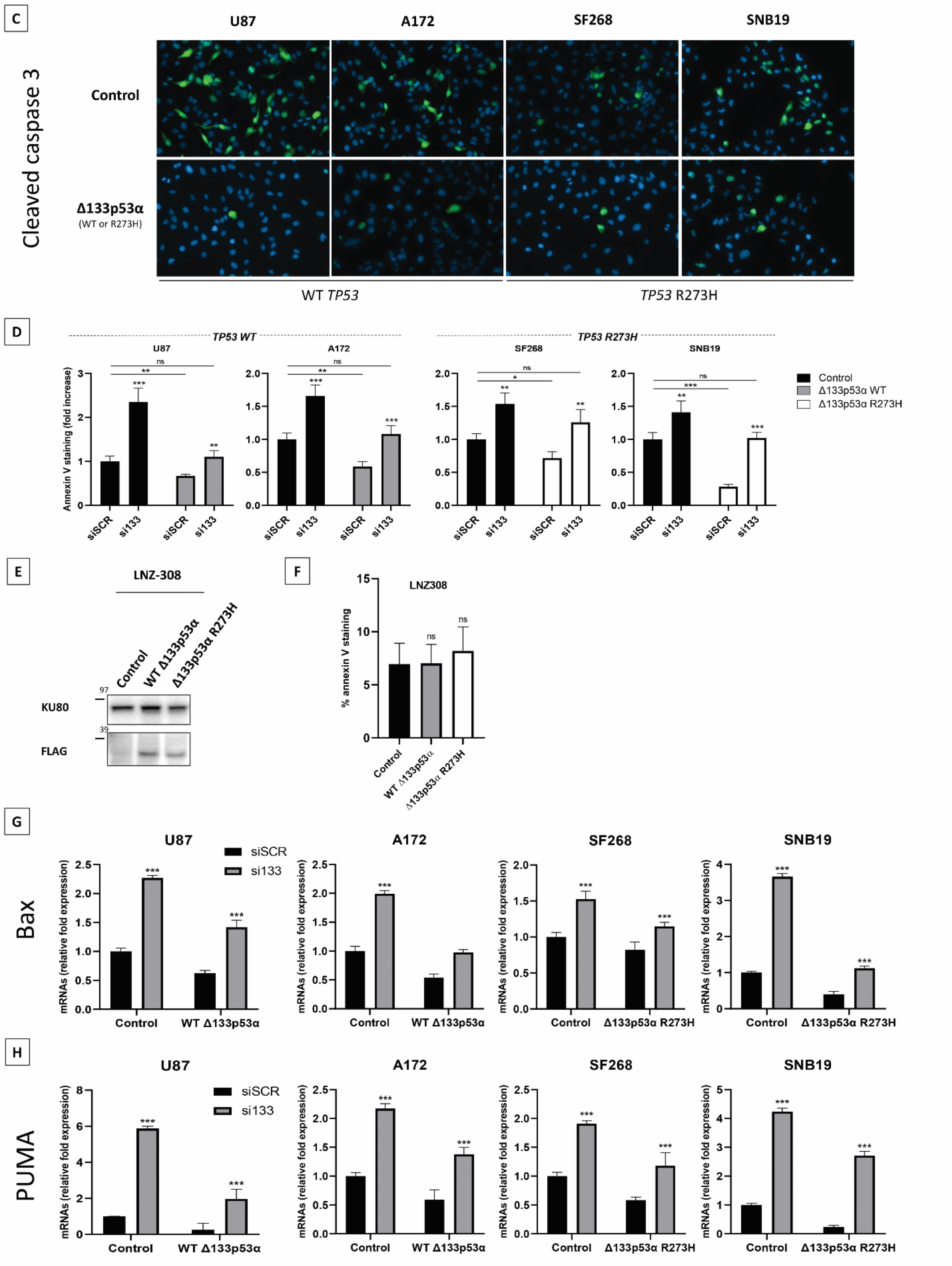
C
Cleaved caspase 3
U87
A172
SF268
SNB19
Control
Δ133p53α
(WT or R273H)
WT TP53
TP53 R273H
D
TP53 WT
TP53 R273H
U87
A172
SF268
SNB19
Annexin V staining (fold increase)
siSCR
si133
ns
*
**
***
Control
Δ133p53α WT
Δ133p53α R273H
E
LNZ-308
Control
WT Δ133p53α
Δ133p53α R273H
KU80
FLAG
97
39
F
LNZ308
% annexin V staining
Control
WT Δ133p53α
Δ133p53α R273H
ns
G
Bax
U87
A172
SF268
SNB19
siSCR
si133
mRNAs (relative fold expression)
Control
WT Δ133p53α
Δ133p53α R273H
***
H
PUMA
U87
A172
SF268
SNB19
siSCR
si133
mRNAs (relative fold expression)
Control
WT Δ133p53α
Δ133p53α R273H
***

Fig. S2: Mutant Δ133p53α R273H fails to promote DNA repair but retains anti-apoptotic functions. **A)** RAD51 mRNA expression measured by Taqman in cells where Δ133p53 isoforms were knocked down by siRNA. Δ133/Δ160 mRNAs knock-down efficiency was measured in Fig. S1J. n=3. **B)** Immunofluorescence staining (60x magnification) of γ-H2AX (Green) and nuclear stain DAPI (blue). n=3. **C)** Immunofluorescence staining (25x magnification) of Cleaved Caspases 3 (Green) and nuclear stain DAPI (blue). n=3. **D)** Cells were seeded and reverse transfected with siScr (control) or si133. Next day, fresh medium with annexin-V dye was added, and the percentage of annexin-V staining was determined 80 hours after transfection. n=3. **E)** Western blot of the WT and mutant FLAG-Δ133p53α overexpressed in the *TP53*-null cells LNZ308. **F)** LNZ308 cells were grown in the presence of annexin V dye in Incucyte® and the percentage of annexin V staining was determined after 80 hours. n=4. **G and H)** PUMA and Bax mRNA expression was measured by Taqman after Δ133 isoforms were knocked down by siRNA. Δ133/Δ160 mRNAs knock-down efficiency was measured in Fig. S1J. n=3.

Supplementary Figure 3

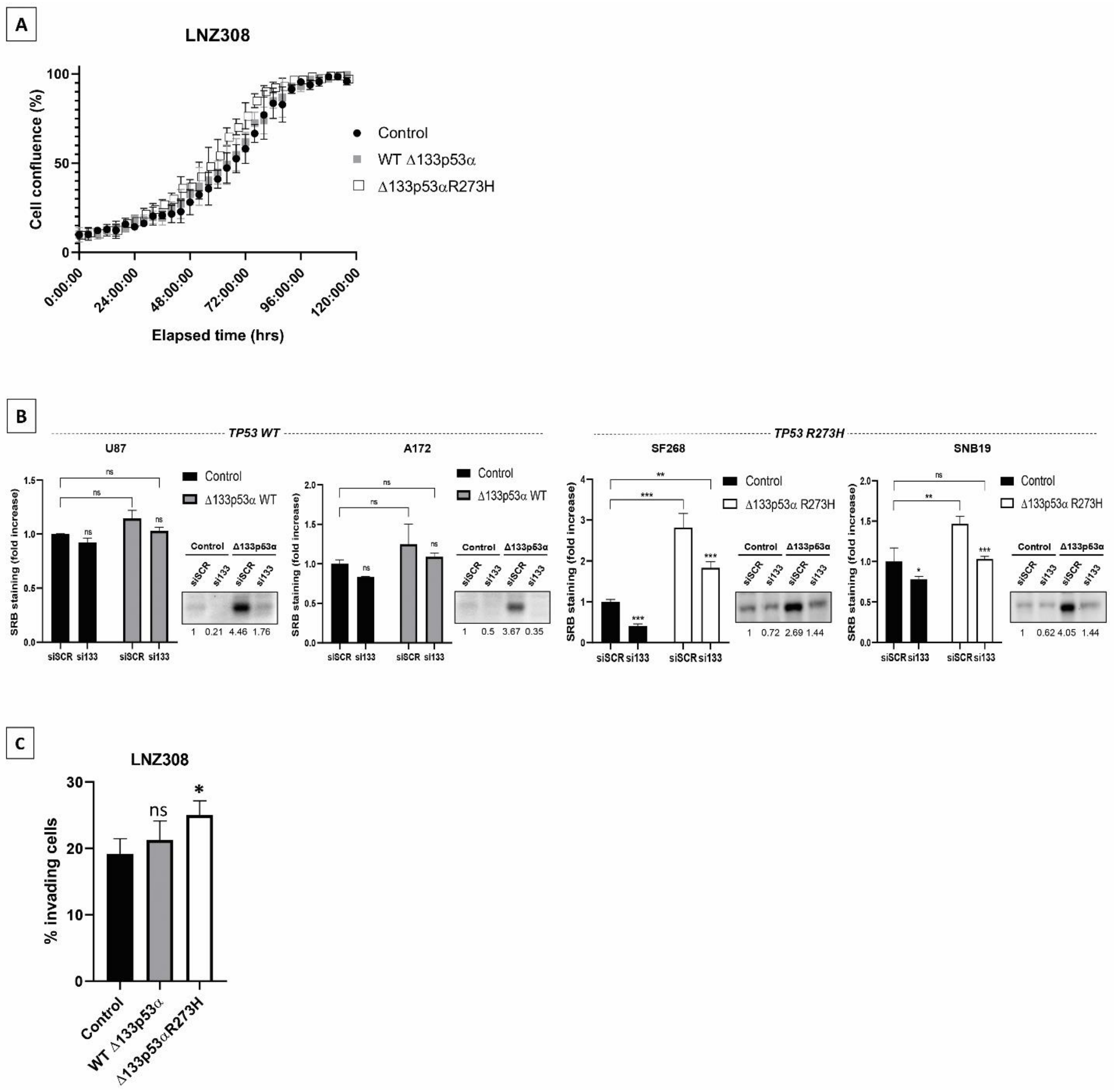

Fig. S3: Mutant Δ133p53α R273H increases cell growth and cellular invasion. **A)** LNZ308 cells were imaged every 4h in incucyte® over 5 days and the percentage of confluence was measured. n=4. **B)** Cells were seeded and reverse transfected with siScr (control) or si133. After 6 days, cells were either used for western blot to assess the efficiency of Δ133p53 knock-down with MAP4 antibody or to determine cell growth by SRB. n=4. **C)** Transwell assay was used to determine the percentage of invading LNZ308 cells 16h after seeding. n=5.

Supplementary Figure 4

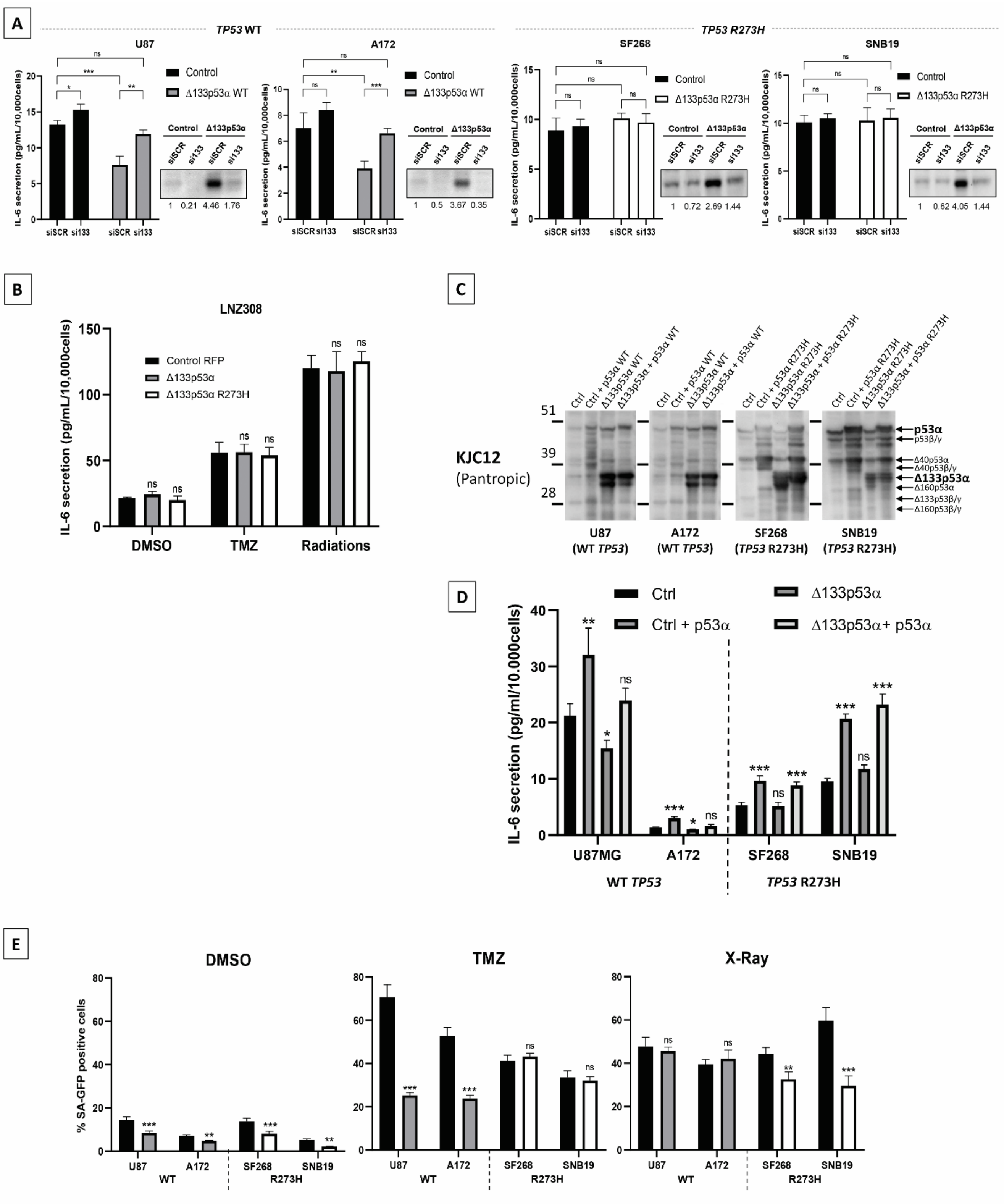

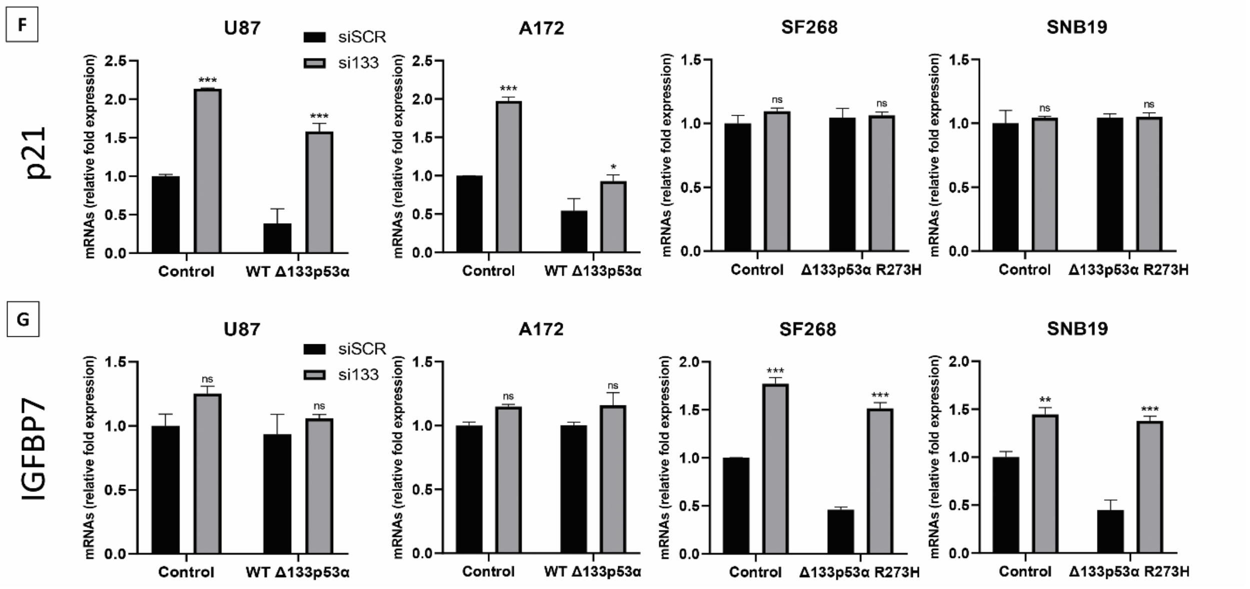

Fig. S4: R273H mutation alters Δ133p53α regulation of cellular senescence, particularly in response to treatment. **A)** Cells were seeded, and reverse transfected with siScr (control) or si133. After 5 days, cells were counted, and proteins were extracted for western blot to assess the efficiency of Δ133p53 knock-down with MAP4 antibody (same images as Fig. 2C and 2D as experiments were made at the same time), and IL-6 secreted in growth media was measured by ELISA. n=4. **B)** IL-6 secreted in growth media was measured by ELISA following LNZ308 cells treatment with DMSO (control), TMZ (50µM for 5 days), or X-rays (10Gy). n=4. **C and D)** WT p53α was overexpressed in the WT cells and p53α R273H was overexpressed in the mutant cell, with and without the overexpression of the corresponding Δ133p53α isoforms. After 5 days, cells were counted and proteins extracted for western blot (C), and IL-6 secreted in growth media was measured by ELISA (D). n=4. **E)** Senescence was determined by measuring the percentage of GFP positive cells in cells treated with DMSO (control), TMZ (50µM for 5 days), or X-rays (10Gy). n=3. **F and G)** p21 and IGFBP7 mRNA expression was measured by Taqman after Δ133 isoforms were knocked down by siRNA. n=3.

Supplementary Figure 5 – Full size western blots

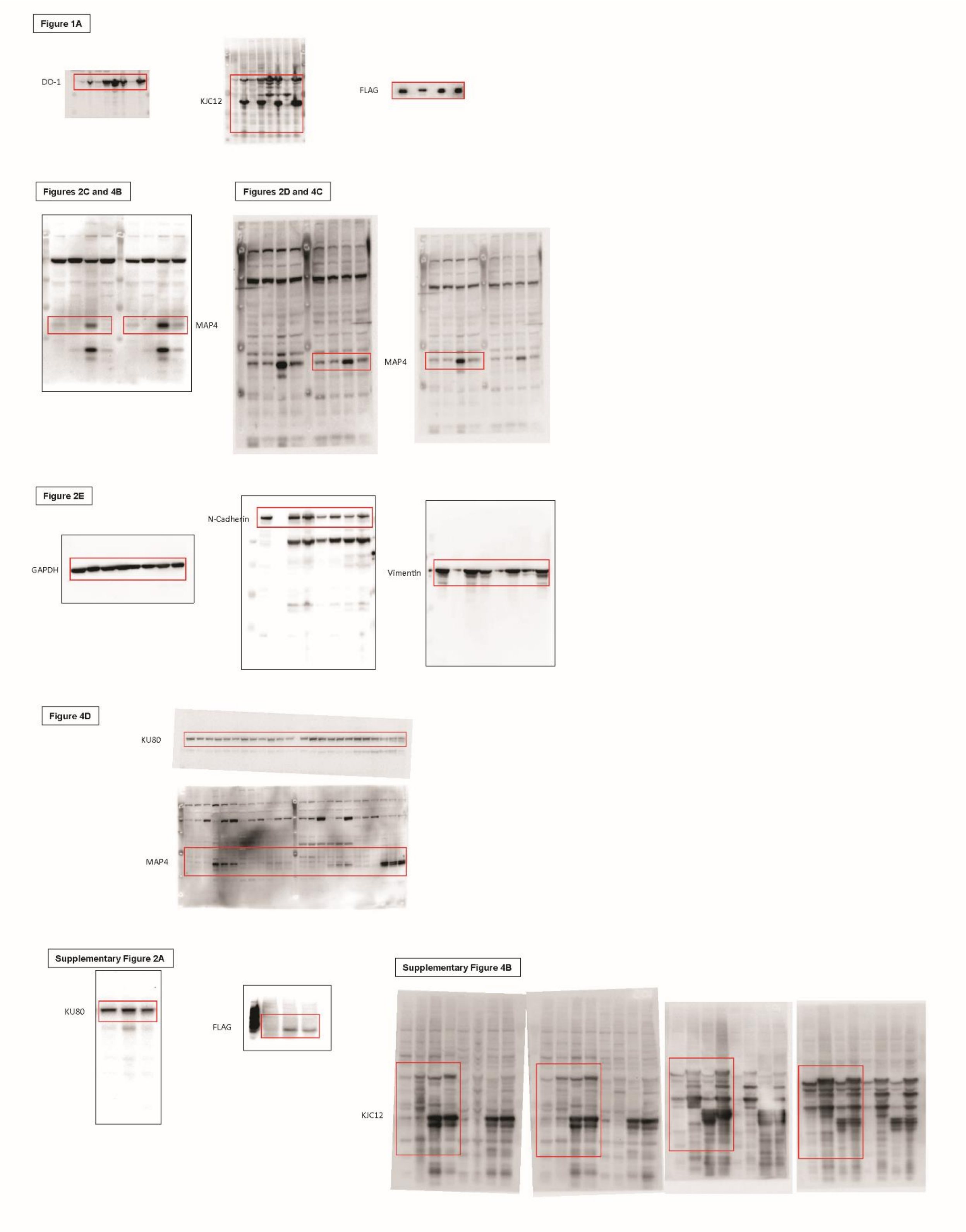

Supplementary tables

Antibody	Source	Catalog	Ref
DO-1	Santa Cruz Biotechnology	sc-126	-
MAP4	Moravian Biotechnologies	-	8
SAPU	Jean-Christophe Bourdon	-	19
KJC12	Jean-Christophe Bourdon	-	20
FLAG-M2	Sigma-Aldrich	F3162	-
N-Cadherin	Cell Signaling Technology	4061	-
Vimentin	Abcam	ab137321	-
GAPDH	MilliporeSigma	ABS16	-
KU80	Invitrogen	MA-1-23314	-
AhR	Invitrogen	MA1-514	-
γ-H2AX	MilliporeSigma	05-636	-
Cleaved-Caspases 3	Cell Signaling Technology	9661S	-

Supplementary Table 1. Antibodies used.

Primer	Sequence	Catalog
IL4I1	-	Hs00541746_m1
IDO1	-	Hs00984148_m1
RAD51	-	Hs00153418_m1
Bax	-	Hs00180269_m1
PUMA/BBC3	-	Hs00248075_m1
p21/CDKN1A	-	Hs00355782_m1
IGFBP7	-	Hs00944483_m1
GAPDH	-	Hs02758991_g1
Δ133 Forward	5′-ACTCTGTCTCCTTCCTCTTCCTACAG-3′	-
Δ133 Reverse	5′-GTGTGGAATCAACCCACAGCT-3′	-

Supplementary Table 2. Primers used.

Supplementary material and methods

Lentiviral particles production

FLAG-Δ133p53α Wild-Type, FLAG-Δ133p53α R273H or a control RFP sequence were cloned into the lentiviral vector pLenti6.3/TO/V5-DEST (Invitrogen) using SpeI and MluI restriction sites. Lentiviral particles were produced using the ViraPower™ Lentiviral Expression System (Invitrogen). pLenti constructs, pLP1, pLP2, and pLP/VSVG plasmids (3µg each) were transfected into 293T/17 Cells (ATCC) in antibiotic-free DMEM using TurboFect (Thermofisher Scientific) and according to manufacturer's instructions. DMEM containing the viral particles was collected 72 hours post-transfection. 1mL DMEM containing FLAG-Δ133p53α Wild-Type, FLAG-Δ133p53α R273H or control-RFP viral particles was added on cells for 24h. Cells were selected with 1µg/ml Blasticidin (Sigma-Aldrich).

Western Blot

Cells were lysed in RIPA (CellSignaling Technology) and quantified using Bradford assay (Biorad). Proteins were supplemented with NuPAGE 4X loading buffer (Invitrogen) and boiled for 5 minutes. Samples were then loaded onto a NuPAGE™ 10% Bis-Tris gel (Novex, Invitrogen) and ran with MOPS buffer (NuPAGE, Invitrogen). Samples were then transferred on 0.2µm nitrocellulose membrane (Amersham) in Tris-Glycine transfer buffer (NuPAGE, Invitrogen). Membranes were blocked in 1:1 mixture of Superblock (Invitrogen) and PBS containing 0.1% Tween-20. Membranes were incubated with the primary antibodies overnight at 4°C, incubated in a mouse, sheep, or rabbit HRP- conjugated secondary antibody (Jackson immunoresearch) at room

temperature for 1 hour and washed twice in PBS-0.1% Tween-20 and once in PBS. ECL (Amersham) developing reagent was applied to detect protein bands with the Biorad imager.

Transcriptome and Analysis

RNA Integrity Number (RIN) was measured by RNA ScreenTape® on TapeStation (Agilent Technologies) by the CCR Genomic Core (Bethesda, MD, USA). Only samples with RIN>9 were used for transcriptome analysis. Library was prepared with the Illumina® Stranded mRNA Ligation Kit and sequencing (40M reads, paired-end 150 bp) was performed on NovaSeq6000 (Illumina) by the CCR sequencing facility (Frederick, MD, USA). Reads of the samples were trimmed for adapters and low-quality bases using Cutadapt before alignment with the reference genome (hg38) and the annotated transcripts using STAR. In addition, the gene expression quantification analysis was performed for all samples using STAR/RSEM tools.
